# Weeds in a Changing Climate: Vulnerabilities, Consequences, and Implications for Future Weed Management

**DOI:** 10.3389/fpls.2017.00095

**Published:** 2017-02-13

**Authors:** Kulasekaran Ramesh, Amar Matloob, Farhena Aslam, Singarayer K. Florentine, Bhagirath S. Chauhan

**Affiliations:** ^1^Indian Council of Agricultural Research – Indian Institute of Soil ScienceBhopal, India; ^2^Department of Agronomy, Muhammad Nawaz Shareef University of AgricultureMultan, Pakistan; ^3^The Centre for Plant Science, Queensland Alliance for Agriculture and Food Innovation, The University of Queensland, ToowoombaQLD, Australia; ^4^Department of Agronomy, University of AgricultureFaisalabad, Pakistan; ^5^Ayub Agricultural Research InstituteFaisalabad, Pakistan; ^6^Centre for Environmental Management, Faculty of Science and Technology, Federation University Australia, Mount HelenVIC, Australia

**Keywords:** weed, climate change, crops, agricultural, management crop, vulnerabilities

## Abstract

Whilst it is agreed that climate change will impact on the long-term interactions between crops and weeds, the results of this impact are far from clear. We suggest that a thorough understanding of weed dominance and weed interactions, depending on crop and weed ecosystems and crop sequences in the ecosystem, will be the key determining factor for successful weed management. Indeed, we claim that recent changes observed throughout the world within the weed spectrum in different cropping systems which were ostensibly related to climate change, warrant a deeper examination of weed vulnerabilities before a full understanding is reached. For example, the uncontrolled establishment of weeds in crops leads to a mixed population, in terms of C_3_ and C_4_ pathways, and this poses a considerable level of complexity for weed management. There is a need to include all possible combinations of crops and weeds while studying the impact of climate change on crop-weed competitive interactions, since, from a weed management perspective, C_4_ weeds would flourish in the increased temperature scenario and pose serious yield penalties. This is particularly alarming as a majority of the most competitive weeds are C_4_ plants. Although CO_2_ is considered as a main contributing factor for climate change, a few Australian studies have also predicted differing responses of weed species due to shifts in rainfall patterns. Reduced water availability, due to recurrent and unforeseen droughts, would alter the competitive balance between crops and some weed species, intensifying the crop-weed competition pressure. Although it is recognized that the weed pressure associated with climate change is a significant threat to crop production, either through increased temperatures, rainfall shift, and elevated CO_2_ levels, the current knowledge of this effect is very sparse. A few models that have attempted to predict these interactions are discussed in this paper, since these models could play an integral role in developing future management programs for future weed threats. This review has presented a comprehensive discussion of the recent research in this area, and has identified key deficiencies which need further research in crop-weed eco-systems to formulate suitable control measures before the real impacts of climate change set in.

## Introduction

To sustain food production for the world’s burgeoning human population (Parry et al., 2005), there is an urgent need to discover vulnerabilities and adaptive measures in managed ecosystems (Howden et al., 2007). It is unequivocal that food security, be either availability, accessibility, utilization, and/or system stability, is dependent on climate (Killman, 2008). Food security is potentially vulnerable to climate change since climate plays a pivotal role in determining growth, development, and perpetuation of all organisms. Climate is defined as the sum of weather conditions of a given area, quantified as long-term statistics of meteorological variables (World Meteorological Organization, 1992). These variables include temperature, wind, precipitation, and sunshine hours, all of which are essential for growth, development, and productivity of vegetation and in turn, human welfare.

In recent decades, changes in climate have caused significant impacts on natural and human ecosystems^1^. These impacts of climate change, irrespective of their cause, illustrate the sensitivity of natural as well as human ecosystems to variations in the function of climatic systems, interaction between its components, or changes in external forces either naturally or due to anthropogenic reasons (IPCC, 1995). Of particular interest here is that agriculture may be jeopardized by climate change (Kang and Banga, 2013; Chauhan et al., 2014), since changes in weather factors have a significant effect on growth of all plant species, including crops and weeds. Rising atmospheric CO_2_ and temperature are expected to pose both direct and indirect consequences for agricultural production, sustainability, water availability and, therefore, food security (Sinha and Swaminathan, 1991; Chauhan et al., 2014). However, in many ways, extremes of weather events associated with climate change are a more serious concern from farmers’ perspectives on crop management as compared with more subtle changes brought by the actual increases in temperature, CO_2_ levels, water availability and associated weather events. To cope with these extreme changes, future development needs to make adjustments in technology, management practices, and legislation (Bhat and Jan, 2010).

At the more subtle level, it is recognized that weeds are aggressive, troublesome, and competitive elements within croplands. Contrary to other pests, weeds share a similar trophic level with crop plants, and by competing for scarce resources they cause enormous crop yield losses. The focus of this paper is on the dynamics of weed-crop competition and how they are influenced by climate, since this has important regional and global implications for food production. For example, the incessant rains during the *kharif* season (June–September) in India have made weed management a challenge, particularly in soybean-based cropping systems. Abrupt changes in climatic variables are likely to result in stressed crop plants, which are vulnerable to attack by insect pests and pathogens (Reddy, 2013), and makes them less competitive against weeds (Patterson, 1995). It is important to note here that this area is extremely complex, as is shown by the work of Chen and McCarl (2001), who found that higher temperatures increase pesticide cost variance for maize (*Zea mays* L.), potatoes (*Solanum tuberosum* L.), and wheat (*Triticum aestivum* L.), while decreasing it for soybeans (*Glycine max* (L.) Merr.). They also reported that rainfall was directly proportional to unit land pesticide usage costs for these crops in the USA.

With a lack of precise information on the effects of climate change on agricultural pests, understanding of this issue remains a major obstacle for remedial measures. The ecological, environmental, and economic costs of not understanding these interactions can be substantial (Ziska and McConnell, 2015). These authors provided a comprehensive review of work done on weeds in a changing climate, with a particular emphasis on the vulnerabilities of crops and cropping systems to weed pressure in changing climate regimes.

## Climate Change and Weeds

The current atmospheric burden of the two most important greenhouse gases (carbon dioxide and methane), are unprecedented (Petit et al., 1999) and have emerged as the greatest ecological challenge of the 21^st^ century (Kang and Banga, 2013). The impact of climate change on weedy vegetation may be manifested in the form of geographic range expansions (migration or introduction to new areas), alterations in species life cycles, and population dynamics. Migration of weeds will subsequently result in a differential structure and composition of weed communities within natural and managed ecosystems. Through the lens of climate change, Peters et al. (2014) outlined three distinct types of shifts in weedy vegetation (range, niche, and trait shifts), occurring at different scales (landscape, community, and population scales), respectively. Changes in weed biology, ecology, and interference potential, in the wake of climate change, will result in complex crop-weed interactions that necessitate alternative adaptive mechanisms. There is a general perception that climate change will result in a differential growth pattern between crops and weeds, as major weeds of the world have the C_4_ pathway and they will become more competitive, although this is certainly not a simple matter due to the adaptive mechanisms in weedy species.

### Weed Response to Increasing CO_2_ Levels

There is an ever-growing consensus that the earth’s climate is changing, and despite the efforts made to reduce CO_2_ emissions, there is an increasing pressure to identify adaptive mechanisms in agro-ecosystems (Howden et al., 2007). The record of atmospheric CO_2_ obtained from Mauna Loa observatory at Hawaii indicated a 20% increase from 311 ppm in the mid-1950s to 375 ppm in 2001 (Keeling and Whorf, 2004), even though Mauna Loa and other global monitoring sites are situated in areas well away from regions of rapid CO_2_ production. Previous studies have quantified a difference of 80 ppm in the CO_2_ concentration between urban and suburban areas (Idso et al., 1988, 2001; Ziska et al., 2001). This observation suggests that although data from Mauna Loa observatory mirrors the global increase, regional increases may be even more substantial due to rapid urbanization and intensive cropping, especially in Asia. This increase will continue in the near future, with estimates suggesting that it may reach 600 ppm (Schimel et al., 1996), while the Inter-Governmental Panel on Climate Change have suggested, as a conservative estimate, 700 ppm by the end of the century (IPCC, 2007).

The projected increase in atmospheric CO_2_ is known to favor net photosynthesis in C_3_ plants (three quarters of global agriculture is represented by C_3_ crops; Kimbal, 1983) by limiting the loss of CO_2_ via photorespiration and increasing the CO_2_ concentration gradient from air to the leaf interior (Ziska, 2000). By contrast, plants with a C_4_ photosynthetic pathway manifest little response to elevated CO_2_ as they have an internal mechanism to concentrate CO_2_ at the site of CO_2_ carboxylation. Thus, from this perspective, ongoing increase in the atmospheric CO_2_ concentration will have crucial implications for weed-crop competition and crop yield losses. Numerous studies have addressed weed-crop interactions by evaluating the comparative growth and physiology of C_3_ crops and C_4_ weeds, and concluded that an elevated CO_2_ concentration generally favors the vegetative growth of C_3_ plant species over those with C_4_ pathways (Patterson, 1995). However, not all crops are based on C_3_ pathways, and not all weeds are C_4_ based (Ziska et al., 2010). Hence, while the above concept is relevant for C_3_ cereals such as rice, which compete, in the main, with C_4_ grassy and broad-leaved weeds, this situation is not universal. There are many C_4_ crops of economic significance, such as maize, sugarcane, and sorghum, which have competition from important C_3_ weeds, for example, *Chenopodium album* L. (Ziska, 2000). This implies that weed-related yield losses of C_4_ crops will tend to increase under elevated CO_2_, but this will not occur with C_3_ crops, as elevated CO_2_ will be a crucial factor in realizing the potential benefits of CO_2_ fertilization.

Notwithstanding this understanding, the abundance and appearance of weeds varies according to regions, crops, and management systems, which complicates management approaches. For example, *Phalaris minor* Retz., a C_3_ species, is a problematic weed in wheat in the Indo-Gangetic Plains of North India. The C_3_ weeds in other areas include *Avena fatua* L., *Chenopodium album* L., *Cirsium arvense* (L.) Scop., *Convolvulus arvensis* L., and *Ludwigia hyssopifolia* (G. Don) Exell. Weedy rice (*Oryza sativa* L.) is also a C_3_ weed in rice in many Asian countries, including Vietnam, Sri Lanka, Malaysia, Thailand, the USA, and the Philippines (Chin et al., 2013; Chauhan, 2013). The third important grain crop, maize, is a C_4_ species, and as noted, although there were some variations in the experimental conditions, C_3_ species generally responded more favorably than C_4_ species to an increased concentration of CO_2_ (Patterson, 1995). Of further interest is that Ziska and McClung (2008) indicated a greater physiological plasticity and genetic diversity among weedy (red) rice relative to cultivated rice, which may impact weed-crop competition with increased atmospheric CO_2_.

It is becoming clear that predicting competitive outcomes based on species grown in isolation, may not adequately quantify crop-weed competition as a function of increasing CO_2,_ as weeds usually occur in a mixture (Ziska, 2001). Therefore, there is a need to evaluate the effects of weed competition on crops in an environment of mixed of weeds and crops since most of the studies on the effect of CO_2_ on crops and weeds have included weed and crop species in isolation. Only a few studies have examined the response of crops and weeds to CO_2_ in competitive environments (Ziska, 2004) and a very little attention has been given to the effect of elevated CO_2_ on the geographical distribution of weeds in managed ecosystems (McDonald et al., 2009). In a study by Ziska et al. (2010), an increase in CO_2_ concentration resulted in a significant decrease in plant relative seed yield of a cultivated rice (C_3_ crop species) variety, but the reverse for a weedy rice biotype, also a C_3_ species. It is thought that this was probably due to a greater physiological plasticity and genetic diversity between a wild and cultivated lines (Treharne, 1989).

Ziska et al. (2011) opined that the increase in atmospheric CO_2_ might also change the biology of invasive weeds. Presumably, an increase in CO_2_ concentration stimulated growth and development of many invasive plant species, for example *Cirsium arvense* L., an invasive perennial C_3_ weed species, had registered a 70% increase in growth with elevated CO_2_ (Ziska et al., 2011). The authors suggested that increasing CO_2_ levels may also increase wind dispersal of weed seeds by either increasing the height of the weed plant or by increasing the plant size. Some of these wind-dispersed invasive weed species are *Cirsium arvense. Sonchus arvensis* L., *Sonchus oleraceus* L., and *Carduus nutans* L.

Changes in weed communities in response to crop establishment methods, wet or dry moisture conditions, tillage regimes, and other management practices are well established in the literature (Nichols et al., 2015; Ramesh, 2015). Nevertheless, very few studies have focused on changes in weed communities in the backdrop of elevated CO_2_ (Koizumi et al., 2004). Variations in the weed competitiveness response to elevated CO_2_ among diverse lines of rice may necessitate screening of vulnerable and resistant cultivars for wider adoption. Although rice could benefit from CO_2_ fertilization, the greater response of its wild relatives, particularly the weedy biotypes of rice, could offset the associated benefits and competitive outcomes as crop yield losses could increase (Ziska, 2008).

Herbicide efficacy may also decrease as CO_2_ concentrations increase (Ziska and Teasdale, 2000; Ziska et al., 2004). An increase in CO_2_ induces morpho-physiological and anatomical changes in plants affecting the rate of uptake and translocation of herbicides (Ziska and Teasdale, 2000; Manea et al., 2011). C_3_ plants showed a decrease in stomata number and conductance and increased leaf thickness, which might interfere with foliar uptake of herbicides (Nowak et al., 2004; Ainsworth and Long, 2005), as well as an increase in starch accumulation on the leaf surface (Patterson, 1995). Moreover, perennial weeds may become even more noxious, if vegetative growth is stimulated as a result of increased photosynthesis in response to elevated CO_2_. These changes are expected to reduce the efficacy of the most commonly used herbicides, such as glyphosate, due to a dilution effect, although the precise mechanism conducive to increased tolerance to glyphosate remains elusive (Manea et al., 2011). This could be due to less translocation as the root system becomes vigorous. In addition, an increase in the root-shoot ratio may play a critical role in herbicide efficacy (Ziska et al., 2004). The authors concluded that CO_2_-induced increase in root biomass could make perennial weeds harder to control in a higher CO_2_ environment. Thus, research is needed to assess the comparative growth and physiological response of C_3_ and C_4_ weeds of different age groups (seedlings or mature plants), and their molecular and biochemical bases to herbicide tolerance under ambient and elevated CO_2_ concentrations. Allocation of resources to below ground parts, source-sink relationships, and mitochondrial respiration also need to be reassessed in the wake of climate change scenarios.

### Weed Response to Elevated Temperature

Atmospheric temperature is regarded as an important indicator of weed species distribution in a geographical area (Patterson et al., 1999). Its rise could alter weed proliferation and competitive behavior in weedy vegetation as well as in crop stands. The indicated likely climate change may favor C_4_ over C_3_ weeds (Tubiello et al., 2007), since under conditions of elevated CO_2_, reduced CO_2_ solubility, and decreased affinity of RUBISCO for CO_2_ would deter C_3_ photosynthesis (Patterson, 1995). As a result, a variation in weed distribution will affect the world’s most important cropping systems, for example, rice-based cropping systems through weed shifts to high latitudes and altitudes. In addition, *Striga* spp. might extend their range to moderate climatic zones (Mohamed et al., 2006, 2007). However, *Striga asiatica* (L.) Kuntze is relatively insensitive to temperature, and changes in the geographical range of the host plants seem to play a critical role in its distribution rather than the direct effects of temperature (Patterson et al., 1982). If this concept is generalized for all parasitic weeds in the Orobanchaceae (Phoenix and Press, 2005), these weeds could pose a serious threat to global crop production, especially in fodders, in the near future. Many C_4_ weeds, such as *Amaranthus retroflexus* L., *Setaria* sp., *Digitaria* sp., *Sorghum halepense* (L.) Pers., and *Paspalum dichotomiflorum* (L.) Michx., are expected to expand further north (Weber and Gut, 2005; Clements and DiTommaso, 2011), which would have a more pronounced effect in the northern Europe, where the number of weed species is lower than in the south (Fried et al., 2010). Milder and wetter winters would tend to increase the survival of winter annual weeds, while thermophile summer annuals will grow more profusely in areas with warmer summers under prolonged growing seasons, enabling them to grow further north (Walck et al., 2011; Hanzlik and Gerowitt, 2012).

Patterson (1995) predicted that climate change would spread arable weed species. For example, *Datura stramonium* L., which needs high temperature for profuse growth (Cavero et al., 1999), would become a more competitive candidate under the climate change scenarios. Warm temperature regimes augmented the abundance of *Hieracium aurantiacum* L. in Australia through accelerated growth and reproduction (Brinkley and Bomford, 2002).

Under warmer conditions, *Setaria viridis* (L.) P. Beauv. germinated later in the (August) season (Dekker, 2003). This was a beneficial temporal non-synchrony with emergence of a maize crop, avoiding crop-weed competition. In contrast, a recent study indicated that this species would be a problematic weed in maize-based cropping systems elsewhere, through synchrony with maize emergence, which is probably due to stimulation by increased temperature (Peters and Gerowitt, 2014). Therefore, *S. viridis* would become a competitor of maize at enhanced temperatures at the time of emergence. This has implications for the northern part of the Central Europe, where temperatures are still below the optimum for this species (Walck et al., 2011). Similarly, *Rottboelliia cochinchinensis* (Lour.) Clayton could invade the central Midwest of the USA and California from Gulf Coast states, with a 3°C rise in temperature (Patterson, 1995). If new weeds are introduced into a non-native area, new and effective herbicides may be needed.

Lee (2011) opined that increased temperature had a greater effect on plant phenological development than elevated CO_2_. The author observed that increasing temperature by 4°C advanced the emergence timing of *C. album* and *S. viridis* by 26 and 35 days, respectively, and flowering time by 50 and 31.5 days. Increased temperatures strongly affected the biomass accumulation by annual grass species during their reproductive phase as compared with the vegetative phase, and such effects are more pronounced in C_3_ than C_4_ plant species. However, the increased temperature was believed to offset potential benefits of elevated CO_2_ by reducing the reproductive output. The uptake and translocation of herbicides in plants and their persistence in soil will also be affected by rising temperatures (Rodenburg et al., 2011). In addition to these effects, temperature will also affect the rate of water absorption and movement, which affects the rate of leaf development, cuticle thickness, and stomatal number and their aperture, thus indirectly affecting herbicide selectivity and efficacy (Bailey, 2004; Chandrasena, 2009; Rodenburg et al., 2011).

### Weed Response to Variation in Rainfall and Drought Spells

A variation in rainfall pattern and increased aridity consistent with a warming climate, could alter weed distribution and their impact on crop production. In the near future, aridity is expected to increase in many agronomically important areas, since an anticipated increase in temperature (1–5°C) is expected with each doubling of the atmospheric CO_2_ level. Rising temperatures also causes greater evaporation, and global trends in rainfall variability suggest that the monsoon regions will become drier (Giannini et al., 2008), leading to a 5–8% increase in drought-susceptible areas (Rodenburg et al., 2011). Trends in future rainfall prediction are difficult to predict, except to forecast more erratic rainfall and consequently, drought and flood would become recurrent phenomena. Under such a scenario, the distribution and prevalence of weeds will be problematic in crop ecosystems, and in particular summer droughts will affect weed management in spring-sown crops (Peters and Gerowitt, 2014). Rodenburg et al. (2010) postulated that under prolonged drought spells, C_4_ and parasitic weeds like *S. hermonthica* will thrive better. Under excess water environments, weeds such as *Rhamphicarpa fistulosa* (Hochst.) Benth. will be favored. A change in rainfall patterns would favor hydromorphic weeds while prolonged drought spells will benefit C_4_ over C_3_ weeds. Under rainfed or dryland environments, little or no rainfall will hamper adequate land preparation for wet season rice as a result of limited water availability for flooding, especially early in the season when the rice is most susceptible to weed competition. This will limit traditional weed management in flooded rice and necessitate the use of herbicides. Rice yield losses are expected to be higher under such circumstances. Asch et al. (2005) emphasized that drought-tolerant rice cultivars would be required to prevent water stress-induced yield losses and to increase rice competitiveness against weeds under rainfed conditions. A change from transplanting to direct seeding of rice, in relation to water saving in South Asia, has already resulted in increased weed competition and changed weed dynamics (Matloob et al., 2015a).

Competition of cotton with *Abutilon theophrasti* Medic. and *Anoda cristata* (L.) Schltdl. increased under drought conditions (Patterson and Highsmith, 1989). A yield reduction due to *Xanthium strumarium* L. was more pronounced in well-watered soybeans compared with water-stressed soybeans (Mortensen and Coble, 1989). An increase in rainfall provided greater competition to wheat growth and yield against *C. arvense* (Donald and Khan, 1992). According to Patterson (1995), weed competition had little effect on crops under water deficit conditions, as the potential crop yield was already reduced by water stress. This was confirmed by Chauhan and Abugho (2013), who showed that rice could not survive under water stress conditions. By contrast, *Amaranthus spinosus* and *Leptochloa chinensis* (L) Nees survived under water stress conditions and produced a significant number of tillers/branches and leaves even at the lowest soil water content.

Increased rainfall frequency and intensity will have an adverse effect on uptake, retention, and activity of soil-applied herbicides (Bailey, 2004; Rodenburg et al., 2011). Increased cuticle thickness and leaf pubescence in response to drought, will reduce herbicide entry into leaves (Patterson, 1995). These attributes can also affect growth and recovery of crops and weeds following herbicide application. Increasing aridity and drought will increase herbicide volatilization, and, moreover, frequent rain showers will reduce the “rain safe periods” available for herbicide application in a given cropping system posing multidimensional challenges for weed management. An unprecedented increase in rainfall (either as a single rain event or annually) may promote leaching of soil-applied herbicides, and subsequent ground water contamination (Froud-Williams, 1996). A general conclusion that can be drawn from the above discussion is that an increase in rainfall would lead to additional weed pressure, thus increasing the herbicide costs and overall cost of production of major crops.

### Weed Response to the Interactive Effects of Climatic Variables

Climate change causes extinctions and alters species distributions of flora and fauna, and exerts inescapable impacts on various antagonistic and mutualistic interactions among terrestrial species (Tylianakis et al., 2008). As noted earlier, the conventional concept that CO_2_ enrichment favors C_3_ plant species over C_4_ by stimulating net photosynthesis, is modified by other associated climate variables affecting this (simple) response (Prior et al., 2003; Hikosaka et al., 2005). The interactive effect of the CO_2_ enrichment will affect weed-crop competition simultaneously or sequentially in a complex manner, quite differentially from its effect on the photosynthetic pathway alone. Past research on climate change has focused on manipulating the plant response to the CO_2_ concentration and not on the associated increases in temperature or drought (Bunce and Ziska, 2000; Fuhrer, 2003). These anticipated changes in temperature and moisture projected under changing climates (IPCC, 2007) have obvious implications for germination and the spatial and temporal emergence of weed seeds and seedlings, which require more holistic investigation. For example, dormancy, which is considered one of the major constraints to weed emergence, is expected to be broken earlier or sooner due to greater moisture availability and warmer temperatures (Ooi et al., 2014; Jaganathan and Liu, 2015). Dormancy cycles observed in some species are known to be regulated mainly by soil temperature in temperate environments where water is not seasonally restricted (Batlla and Benech-Arnold, 2004), irrespective of their CO_2_ response. Wand et al. (1999) and Ward et al. (1999) demonstrated that the combined effects of soil water and nutrient stress limited the response of C_3_ plants to elevated CO_2_, but not C_4_ species. Belote et al. (2003) suggested that water availability is a crucial factor that mediates species and community responses to rising CO_2_ concentrations. Information regarding the interactive effects of elevated CO_2_ with sub-ambient temperatures in either C_4_ weeds or crops is scarce. Few studies are available that have examined the growth and reproductive response of C_3_ weeds to the combined effect of temperature and CO_2_. Weeds like *Senna obtusifolia* and *Anoda cristata* exhibited a higher range of growth stimulation under elevated CO_2_ and temperature, however, other weeds (*Triticum repen* L. and *Abutilon theophrasti*) did not respond similarly (Patterson et al., 1988; Tremmel and Patterson, 1993). Moreover, differential enhancement of C_3_ crops and weeds by elevated CO_2_ at sub-optimal temperatures should receive attention. Alberto et al. (1996) found that competitiveness of a C_3_ crop species (rice) relative to a C_4_ weed species (*Echinochloa glabrescens* Munro ex Hook.f.) could be enhanced by elevated CO_2_ alone, but a simultaneous increase in CO_2_ and temperature still favored the weed. Remarkably, the interactive effect of CO_2_ with water availability has been exclusively studied for crop species (Tyree and Alexander, 1993; Bunce, 2004), with little emphasis placed on quantifying differences between crops and weeds of the same photosynthetic pathway. Patterson (1986) found that rising CO_2_ levels favored the growth of both a C_3_ crop (soybean) and C_4_ weeds [*Echinochloa crus-galli* (L.) P.Beauv., *Eleusine indica* (L.) Gaertn., and *Digitaria ciliaris* (Retz.) Koeler)] by improving water-use efficiency under drought, although greater growth stimulation in the C_3_ crop was expected. Studies reporting the interactive effects of rising CO_2_ levels, drought, and weed-crop competition are not common. It could be speculated that if CO_2_ decreases, the water requirement of C_4_ weeds relative to C_3_ crops, C_4_ weeds could still potentially compete successfully with C_3_ crops under a high CO_2_/drought situation (Knapp et al., 1993). Ironically, few studies have focused on how CO_2_-induced changes in phenological development could be modified by other climatic factors such as water supply and/or temperature (Springer and Ward, 2007).

The opinion of Rosenzweig and Hillel (1998) that rising temperature and CO_2_ levels could make crop plants less competitive with weeds, together with a similar prediction a decade later by Wolfe et al. (2008) that weeds would benefit more than cash crops, were both found to be true. *Amaranthus retroflexus* produced more seeds in barley cropping, albeit the growth of barley as well as the weed was reduced in southern Finland (Hyvonen, 2011).

Patterson and Flint (1982) found growth stimulation in soybean and two associated weeds [*Senna obtusifolia* (L.) H. S. Irwin and Barneby and *Crotalaria spectabilis* Roth] with increasing CO_2_ to 675 ppm in Hoagland’s solution. Zhu et al. (2008), while investigating the effect of nutrient and CO_2_ on weed-crop competition using a C_3_ crop (rice) and a C_4_ weed (*E. crus-galli)* model system, found a proportionate increase in rice biomass compared with *E. crus-galli* (in response to 200 ppm increase in CO_2_) under an optimum nitrogen supply. In contrast, at a sub-optimum nitrogen level, elevated CO_2_ reduced the competitive ability of rice against *E. crus-galli*. Hence, an increase in atmospheric CO_2_ will exacerbate rice yield losses under low soil nitrogen status, owing to C_4_ weed competition. Systemic investigations are needed to appraise the interactive effects of key environmental variables on different weed species and communities under diverse ecosystems.

Several modeling studies do not account for the impacts of an increased climatic variability (Tubiello et al., 2007; Orlandini et al., 2008) which is a necessity in weed-crop interactions under the climate change scenario. It is a general perception that the only role of the endogenous process has been emphasized in the weed population dynamics models, which ignores exogenous variables such as climate (Lima et al., 2012). The authors considered that the population dynamics of weeds are a function of ecological interactions within and between plant populations, nutrient and water limitation, rainfall, temperature and stochastic forces. Using a reproduction function (*R*-function), they concluded that interactions between endogenous and exogenous factors are important for management of weed and invasive plants and climate change mitigation. Since predicting the impact of a weed under cultivated conditions at local scale requires a process-based modeling approach integrating local environmental conditions with the differential responses of the crop and weeds, Stratonovitch et al. (2012) have developed a simulation model for winter wheat and a competing weed *Alopecurus myosuroides in UK.*

### Herbicide-Climate Interactions

Herbicide effectiveness is dependent on the local climate/microclimate, and herbicides are no exceptions, particularly foliage applied post-emergence (Kudsk and Kristensen, 1992). A rise in temperature will increase volatility of certain herbicides such as trifluralin, rendering it less efficient. In the 1970s, rapid volatilization of surface-applied alachlor, butachlor, and propachlor occurred from continuously moist soils exposed to a constant 21°C (Beestman and Deming, 1974). Temperatures (day/night 32/22°C and 26/16°C) were not as critical as that of relative humidity in influencing acifluorfen (diphenylether group) phytotoxicity on *Xanthium strumarium* L. and *Ambrosia artemisiifolia* L. (Ritter and Coble, 1981). However, temperature had a significant effect on the degradation of imazapyr (imidazolinone group), flumetsulam (sulfonanilide family), and thifensulfuron (sulfonylurea group) in soil (Mcdowell et al., 1997). Glyphosate absorption is dependent on the atmospheric temperature, as evident from *Desmodium tortuosum* (Sw.) DC., a C_3_ weed (Sharma and Singh, 2001). An increase in temperature or relative humidity increased the efficacy of mesotrione on *X. strumarium* and *A. theophrastii* three-fold (Johnson and Young, 2002). The efficacy of the herbicide pyrithiobac (pyrimidinylthiobenzoic acid group) on *Amaranthus palmeri* L. was reduced at temperatures outside the range of 20-34°C (Mahan et al., 2004). Anderson et al. (1993) found that relative humidity had the most significant effect on the phytotoxic action of glufosinate-ammonium, since this is attributed to changes in cuticle hydration and droplet drying (Ramsey et al., 2005). Studies under controlled environmental chambers in Australia using varying night/day temperatures of 5/10, 15/20, and 20/25°C showed that *Raphanus raphanistrum* L., grown under cooler temperatures of 5/10°C, was poorly controlled with 1,200 g ai ha^-1^ of glufosinate. By comparision, 100% mortality was achieved under 15/20 and 20/25°C for the same dose (Kumaratilake and Preston, 2005), suggesting enhanced efficacy of glufosinate under enhanced atmospheric temperature.

## Implications of Climate Change for Weed-Crop Interactions

Uncertainty in agricultural productivity under a climate change scenario, can be the result of plant-plant interactions through direct effects of a change in temperature and atmospheric CO_2_, or indirect effects at the system level through shifts in crop–weed interactions (Fuhrer, 2003) and other biotic stresses.

### Shifts in Weed Abundance, Distribution, and Competitive Balance

Under ambient conditions, water availability and temperature are the principal determinants of species distribution (Patterson et al., 1999), but there is the recent addition to this list of CO_2_ concentrations through the lens of climate change (Patterson, 1995; Chauhan et al., 2014). The changing climate variables may either increase the distribution range of weed species in response to a change in atmospheric temperature, or allow some non-potent weeds to dominate weed abundance as crop-weed interactions may increasingly favor C_3_ weeds (Bazzaz et al., 1985). Other than geographical distribution, the projected climate change might impact their population biology (Patterson et al., 1999; Ziska and Goins, 2006), causing them to move to new areas lying at higher altitudes and latitudes (Patterson, 1995; Ziska and Dukes, 2011). Such effects have been proposed for *Striga* sp., which are expected to extend their geographic range (Mohamed et al., 2006). Climate change will alter the distribution of plant species and overall functioning and productivity of ecosystems. For example, increased abundance of woody vines as a consequences of rising CO_2_ levels has been associated with an increased tree mortality and reduced tree regeneration in forests throughout the world (Phillips et al., 2002). Similarly, an increase in parasitic weeds would become a serious threat to productivity of rice and sorghum crops under rainfed agriculture (Rodenburg et al., 2011). According to Holm et al. (1997), most of the troublesome C_3_ and C_4_ weeds of the arable land are limited to tropical and subtropical regions, primarily due to low temperatures at higher latitudes. Preliminary data showed an increased tolerance in many weeds to low temperatures under elevated CO_2_ (Boese et al., 1997), which suggests the possibility of polar-ward expansion for many weed species (Bradley and Mustard, 2005; McDonald et al., 2009; Ziska and Dukes, 2011).

Species either have to adapt *in situ* to new climatic conditions or undergo shifts in their distribution to more favorable locals. McDonald et al. (2009) proposed that if climate change forecasts are realized, damaging endemic weed species of major cropping systems might experience a significant transformation in their host range, besides an overall increase in the chance of invasion by exotic invasive weed species. Besides agronomic weeds, there are also certain non-native weeds whose introduction to new areas can pose ecological and environmental hazards (Mooney and Hobbs, 2000). Several studies have demonstrated that such weeds often benefit from carbonaceous fertilization (Polley et al., 2002; Belote et al., 2003; Ziska and George, 2004). It is believed that under a climate change scenario, these invasive plants would be able to extend their geographic range as well as spread to new areas, including currently agriculturally productive regions (Ziska and Dukes, 2011). An expansion in the geographic range proposed for weeds such as *Lonicera semperviens* L. and *Pueraria lobata* (Lour.) Merr. in the past has now become a reality (Patterson, 1995). Range expansion of arable and invasive weeds in connection with climate change must be studied as an integral part of crop-weed interactions.

In a composite stand of weeds in a cropped field (C_3_ and C_4_ plants), dynamics in insurgence and shifts of the weed populations in favor of specific species is expected over time (Das et al., 2012). These authors further argued that climate change is likely to trigger differential growth in crops and weeds and will have significant implications for weed management across crops and cropping systems. The abundance, competitive ability, and survival of perennial weeds are expected to be higher, since a rise in CO_2_ stimulates tuber and rhizome growth (Chandrasena, 2009). Climate change will result in a greater frequency of extreme weather events such as frequent droughts and cold spells, so that weeds with less phenotypic plasticity may experience population declines (Peters et al., 2014). Lack of rainfall and prolonged drought will limit growth of arable crops and pastures, resulting in a lack of vegetation cover and bare ground, thus allowing invasion by more resilient drought-tolerant weeds. Increasing CO_2_ could alter the competitive balance in a weed-crop mixture through its effect on photosynthesis and stomatal physiology, which is linked with the competitive balance between crops and weeds in a cropping system (Alberto et al., 1996). The range of growth stimulation in response to elevated CO_2_ needs to be determined for both crops and weeds with contrasting carbon fixation pathways, growing in variable densities and species compositions. Under conditions of higher temperature and drought, C_4_ weeds such as *A. retroflexus* tend to dominate C_3_ crops (e.g., soybean). The infestation of *P. minor* is expected to worsen in wheat fields with CO_2_ increase (Mahajan et al., 2012). Likewise, weedy rice will compete more strongly with cultivated rice (Ziska et al., 2010). Exploring differential mechanisms and responses that govern the success of weeds to invade new areas/cropping systems and their ability to utilize growth resources, will be helpful in understanding the implications of rising CO_2_ levels on plant-plant interactions. This also requires characterizing their damage niche (McDonald et al., 2009).

### Effectiveness of Weed Management and Adoption of Best Agronomic Practices

Climate change will indirectly affect the adoption and success of weed management strategies. Looming water crises have been recognized as a major threat to agricultural productivity (Sandhu et al., 2012) notably in irrigated rice (Soomro, 2004; Farooq et al., 2011) with long-term consequences for regional and global food security (Braun and Bos, 2005; Seck et al., 2012). Water requirements of irrigated rice are approximately 2–3 times higher than for any other upland cereal (Bouman et al., 2007; Bouman, 2009; Pathak et al., 2011). Aerobic rice is a potential water-use efficient production system, but a high weed infestation (up to 90% yield reduction; Gowda et al., 2009) has threatened its sustainability, which demands efficient and cost-effective weed management techniques (Anwar et al., 2012). Frequent drought spells and erratic rainfall will affect productivity and sustainability of upland and low land rice production systems. There will be a trade-off between water-use efficient rice production methods and weed management. In upland rice, drought tolerance will be needed not only to cope with water scarcity but also to safeguard production losses against weeds by maintaining or improving a competitive edge (Asch et al., 2005). In aerobic or dry-seeded rice, the switch over from transplanting in respect of water saving, induces qualitative and quantitative changes in rice weed flora (Matloob et al., 2015a). The inherent size differential of transplanted rice seedlings in conjunction with flooded environments provided a distinct competitive advantage, i.e., an earlier growth and germination over a wide range of weed species that otherwise are quite problematic in aerobic rice. With a dwindling water supply and more severe drought spells, flooding will not be available as a potential weed management tool in the near future. Hand weeding was 35% higher when the flooding regime was altered from permanent to temporary flooding (Latif et al., 2005). This means that farmers lacking alternate means and resources to combat weeds will suffer significant yield losses. Moreover, dry tillage practices, alternate wetting and drying regimes, and extended periods during which soil is not flooded, will result in the insurgence of non-native and difficult-to-control weeds (Chauhan et al., 2014). Under drought conditions, rice (C_3_) is already a poor weed competitor (Saito, 2010) and will be under greater pressure due to increased competition from C_4_ weeds, which comprise the majority of weed flora infesting rice fields (Caton et al., 2010). In rainfed rice, a lack of rainfall early in wet seasons may compel farmers to adjust their timing of land preparation and subsequent planting. This might affect synchronization of rice sensitive growth periods with emergence and active growth period of troublesome weeds. Hence, it seems that strategies aimed at mitigating climate change effects on crop production like drought-tolerant rice germplasm and water saving rice cultivation, will also have implications for weed management (Rodenburg et al., 2011).

Increasing interest in conservation agriculture has created a reliance on glyphosate for weed management (Shaner, 2000), and the continuous use of this herbicide may result in evolution of resistant biotypes of major weeds. In wheat, resource conservation technologies, such as no-till systems, have emerged as an important breakthrough (Erenstein et al., 2008). However, adoption of no-till approaches which are characterized by minimal soil disturbance, may affect the abundance and floristic composition of weeds (Matloob et al., 2015b). Hardy weeds, such as *Rumex* sp., are expected to be higher in zero-till wheat fields (Chauhan et al., 2014).

Ziska et al. (1999) and Ziska and Teasdale (2000) have shown that herbicides (e.g., glyphosate) will be rendered less effective against weeds under CO_2_ levels anticipated in the near future. Increased tolerance to glyphosate under elevated CO_2_ has been recorded for both agricultural and invasive weed species (Ziska and McConnell, 2015). These alarming findings revealed a sustained increase in photosynthesis and growth of perennial weeds such as *Elymus repens* (L.) Gould. with a concurrent decrease in herbicide efficacy and increased potential of invasion and competition (Ziska and Teasdale, 2000). Differential tolerance to glyphosate exhibited by certain weeds under elevated CO_2_ is also an issue. Whilst the response of weeds such as *A. retroflexus* was not affected by elevated CO_2_, *Chenopodium album* and *Cirsium arvensis* manifested a significant glyphosate tolerance (Ziska et al., 1999; Ziska et al., 2004). A variable response to glyphosate was observed even for invasive grass species possessing the same carbon fixation pathway (Manea et al., 2011). Hence, it can be inferred that some weeds will be more problematic in the near future in glyphosate-tolerant crops or under conservation agriculture. Another difficulty will be the knockdown of perennial weeds if glyphosate efficacy is reduced due to climate change. An increase in rhizome and tuber growth, coupled with an increase in biomass, would cause a dilution effect on any herbicide application, causing an increase in weed control costs. Direct effects of climate change on plant physiology, anatomy, and morphology will indirectly affect herbicide efficacy by influencing uptake, translocation, and metabolism. Changes in physical environments, such as drought spells or prolonged rainy seasons, may limit the field conditions necessary for herbicide applications. Climate change will have implications for all dimensions of chemical weed management including application, spray drift, persistence, metabolism, and herbicide efficacy. This justifies diversifying current weed management tactics as well as the urgency of a sound knowledge regarding the ecology and biology of weeds in a changing climate.

After a catastrophic climatic events such as drought or flood, weeds will have a greater chance to colonize and invade disturbed habitats. Chemical control measures may become less effective due to a change in the external environment (drier and warmer conditions) or changes in anatomy, growth physiology, and phenology of the target weed flora (Chauhan et al., 2014; Ziska and McConnell, 2015). Asexual reproduction through below-ground parts is always conducive to spread, irrespective of water availability. Extremes of moisture availability, viz., flood as well as drought, hinder physical management methods such as hoeing, inter-cultivation, etc. It seems that growers will have to carefully synchronize the timing of their control measures with the weed life cycle since these will also respond to climate change. The opinion of Chandrasena (2009) that adaptive responses should be based on a better knowledge on how plant communities will respond to climate change rather than *ad hoc* responses, is therefore valid in the current context.

## Conclusion and Future Research Needs

Research is needed to unravel whether the so-called CO_2_ fertilization could compensate for other negative effects of climate change on crop-weed competition. Moreover, the response of agricultural and invasive weeds to other climatic factors and associated parameters such as temperature, drought, rainfall, and an extended growing period should be explicitly assessed in conjunction with an anticipated rise in CO_2_ concentration to predict a wider picture of competitive outcomes in managed and natural ecosystems. The effect of climate change on the geographic distribution of invasive weeds will be a subject of interest in the near future. Research efforts are also needed to explore the adaptive mechanisms/practices to facilitate crop production with changing conditions under climate change scenarios and, at the same time, asses their effectiveness, required time span, and economic and ecological costs.

Climate change is a looming global crisis and its impacts on agricultural weeds have not been well explored. Conventional thinking around carbon pathways in plants and nutrient management in crops could partially solve the climate change implications, but weed problems could also be aggravated in the wake of increasing CO_2_ concentration, high temperature, and most significantly by water stress. These conditions might necessitate the adoption of new agronomic practices to enhance weed competitiveness. As crops and weeds share the same trophic level, the stimulatory or inhibitory behavior of the climate variables on crops should generally hold true for weeds. An increase in atmospheric temperature was found to favor weed growth as well as herbicide efficacy. Although there is a dominance of C_4_ weeds in agriculture, C_3_ and C_3_-C_4_ intermediate pathways of prominent weeds would pose severe crop-weed competition in the years to come. Importantly, due to species interaction, there is a need to study all possible combinations of plant-weed carbon fixation pathways, C_3_ crops and C_3_ weeds, C_4_ crops and C_4_ weeds, C_3_ crops and C_4_ weeds, and C_4_ crops and C_3_ weeds, while studying the impact of climate change on crop-weed competitive interactions. Several weeds will exert additional pressure for crop-weed competition under the climate change scenario. More adaptive research studies, including complex research conditions, could yield useful solutions for managing yield reduction in the ensuing decades.

## Author Contributions

BSC developed the initial concept and outline. KR and AM took lead in expanding the content. FA, SKF, and BSC contributed and edited the manuscript.

## Conflict of Interest Statement

The authors declare that the research was conducted in the absence of any commercial or financial relationships that could be construed as a potential conflict of interest.

## Footnotes

1 http://www.ipcc.ch

## References

[B1] Ainsworth E. A. Long S. P. (2005). What have we learned from 15 years of free-air CO2 enrichment (FACE)? A meta-analytic review of the responses of photosynthesis, canopy properties and plant production to rising CO2. *New Phytol.* 165 351–372. 10.1111/j.1469-8137.2004.01224.x 15720649

[B2] Alberto A. M. Ziska L. H. Cervancia C. R. Manalo P. A. (1996). The influence of increasing carbon dioxide and temperature on competitive interactions between a C3 crop, rice (*Oryza sativa*) and a C4 weed (*Echinochloa glabrescens*). *Aust. J. Plant Physiol.* 23 795–802. 10.1071/PP9960795

[B3] Anderson D. M. Swanton C. J. Hall J. C. Mersey B. G. (1993). The influence of temperature and relative humidity on the efficacy of glufosinate-ammonium. *Weed Res.* 33 139–147. 10.1111/j.1365-3180.1993.tb01927.x

[B4] Anwar M. P. Juraimi A. S. Puteh A. Man A. Rahman M. M. (2012). Efficacy, phytotoxicity and economics of different herbicides in aerobic rice. *Acta Agric. Scand. B Soil Plant Sci.* 62 604–615. 10.1080/09064710.2012.681060

[B5] Asch F. Dingkuhn M. Sow A. Audebert A. (2005). Drought-induced changes in rooting patterns and assimilate partitioning between root and shoot in upland rice. *Field Crops Res.* 93 223–236. 10.1016/j.fcr.2004.10.002

[B6] Bailey S. W. (2004). Climate change and decreasing herbicide persistence. *Pest Manage. Sci.* 60 158–162. 10.1002/ps.785 14971682

[B7] Batlla D. Benech-Arnold R. L. (2004). Seed dormancy loss assessed by changes in population hydrotime parameters. *Dev. Predictive Model. Seed Sci. Res.* 14 277–286. 10.1079/SSR2004177

[B8] Bazzaz F. A. Garbutt K. Williams W. E. (1985). “Effect of increased atmospheric carbon dioxide concentration on plant communities,” in *Direct Effects of Increasing Carbon Dioxide on Vegetation*, eds Strain B. R. Cure J. D. (Washington, DC: United States Department of Energy), 168.

[B9] Beestman G. B. Deming J. M. (1974). Dissipation of acetanilide herbicides from soils. *Agron. J.* 66 308–311. 10.2134/agronj1974.00021962006600020035x

[B10] Belote R. T. Weltzin J. F. Norby R. J. (2003). Response of an understory plant community to elevated CO2 depends on differential responses of dominant invasive species and is mediated by soil water availability. *New Phytol.* 161 827–835. 10.1111/j.1469-8137.2004.00977.x 33873709

[B11] Bhat N. H. Jan S. (2010). Impact of climate change on crop productivity: need of adjustment in agriculture. *Res. J. Agric. Sci.* 1 483–486.

[B12] Boese S. R. Wolfe D. W. Melkonian J. (1997). Elevated CO2 mitigates chilling-induced water stress and photosynthetic reduction during chilling. *Plant Cell Environ.* 20 625–632. 10.1111/j.1365-3040.1997.00082.x

[B13] Bouman B. A. M. (2009). How much water does rice use? *Rice Today* 8 28–29.

[B14] Bouman B. A. M. Humphreys E. Tuong T. P. Barker R. (2007). Rice and water. *Adv. Agron.* 92 187–237. 10.1016/S0065-2113(04)92004-4

[B15] Bradley B. A. Mustard J. F. (2005). Identifying land cover variability distinct from land cover change: cheatgrass in the Great Basin. *Remote Sens. Environ.* 94 204–213. 10.1016/j.rse.2004.08.016

[B16] Braun J. Bos M. S. (2005). “The changing economics and politics of rice: Implications for food security, globalization, and environmental sustainability,” in *Proceedings of the World Rice Research Conference Held in Tokyo and Tsukuba*, eds Toriyama K. Heong K. L. Hardy B. (Tsukuba: Japan International Research Center for Agricultural Sciences), 7–20.

[B17] Brinkley T. R. Bomford M. (2002). *Agricultural Sleeper Weeds: What is the Potential Threat?* Kingston, ACT: Bureau of Rural Sciences.

[B18] Bunce J. A. (2004). Carbon dioxide effects on stomatal responses to the environment and water use by crops under field conditions. *Oecologia* 140 1–10. 10.1007/s00442-003-1401-6 14557864

[B19] Bunce J. A. Ziska L. H. (2000). “Crop ecosystem responses to climatic change: crop/weed interactions,” in *Climate Change and Global Crop Productivity*, eds Reddy K. R. Hodges H. F. (New York, NY: CABI), 333–348.

[B20] Caton B. P. Mortimer M. Hill J. E. Johnson D. E. (2010). *A Practical Field Guide to Weeds of Rice in Asia*, 2nd Edn Los Baños: International Rice Research Institute, 118.

[B21] Cavero J. Zaragoza C. Suso M. L. Pard A. (1999). Competition between maize and *Datura stramonium* in an irrigated field under semi-arid conditions. *Weed Res.* 39 225–240. 10.1046/j.1365-3180.1999.00140.x

[B22] Chandrasena N. (2009). How will weed management change under climate change? Some perspectives. *J. Crop Weed* 5 95–105.

[B23] Chauhan B. S. (2013). Strategies to manage weedy rice in Asia. *Crop Prot.* 48 51–56. 10.1016/j.cropro.2013.02.015

[B24] Chauhan B. S. Abugho S. B. (2013). Effect of water stress on the growth and development of *Amaranthus spinosus. Leptochloa chinensis*, and rice. *Am. J. Plant Sci.* 4 989–998. 10.4236/ajps.2013.45122

[B25] Chauhan B. S. Prabhjyot-Kaur Mahajan G. Randhawa R. J. Singh H. Kang M. S. (2014). Global warming and its possible impact on agriculture in India. *Adv. Agron.* 123 65–121. 10.1017/S1751731116002706

[B26] Chen C. C. McCarl B. A. (2001). An investigation of the relationship between pesticide usage and climate change. *Climatic Change* 50 475–487. 10.1023/A:1010655503471

[B27] Chin D. V. Thien T. C. Bi H. H. Nhiem N. T. Son T. T. N. (2013). “Weed and weedy rice control by imidazolinone herbicides in Clearfield paddy in Vietnam,” in *Proceedings of the 4th Tropical Weed Science Conference*, Chiang Mai, 98.

[B28] Clements D. R. DiTommaso A. (2011). Climate change and weed adaptation: can evolution of invasive plants lead to greater range expansion than forecasted? *Weed Res.* 51 227–240. 10.1111/j.1365-3180.2011.00850.x

[B29] Das T. K. Sharma A. R. Pathak H. (2012). “Crop-weed balance studies under climate change,” in *Climate Change Impact, Adaptation and Mitigation in Agriculture: Methodology for Assessment and Application*, eds Pathak H. Aggarwal P. K. Singh S. D. (New Delhi: Indian Agricultural Research Institute), 131.

[B30] Dekker J. (2003). “Evolutionary biology of the Foxtail (Setaria) species-group,” in *Weed Biology and Management*, ed. Inderjit (Dordrecht: Kluwer Academic Publishers), 65–114.

[B31] Donald W. W. Khan M. (1992). Yield loss assessment for spring wheat (*Triticum aestivum*) infested with Canada thistle (*Cirsium arvense*). *Weed Sci.* 40 590–598.

[B32] Erenstein O. Farooq U. Malik R. K. Sharif M. (2008). On-farm impacts of zero tillage wheat in South Asia’s rice-wheat systems. *Field Crops Res.* 105 240–252. 10.1016/j.fcr.2007.10.010

[B33] Farooq M. Siddique K. H. M. Rehman H. Aziz T. Lee D. Wahid A. (2011). Rice direct seeding: experiences, challenges and opportunities. *Soil Till. Res.* 111 87–98. 10.1016/j.still.2010.10.008

[B34] Fried G. Petit S. Reboud X. (2010). A specialist-generalist classification of the arable flora and its response to changes in agricultural practices. *BMC Ecol.* 10 1–11. 10.1186/1472-6785-10-20 20809982 PMC2939635

[B35] Froud-Williams R. J. (1996). Weeds climate change: implications for their ecology and control. *Asp. Appl. Biol.* 45 187–196.

[B36] Fuhrer J. (2003). Agroecosystem responses to combination of elevated CO2, ozone and global climate change. *Agric. Ecosys. Environ.* 97 1–20. 10.1016/S0167-8809(03)00125-7

[B37] Giannini A. Biasutti M. Held I. M. Sobel A. H. (2008). A global perspective on African climate. *Climatic Change* 90 359–383. 10.1007/s10584-008-9396-y

[B38] Gowda P. T. Shankaraiah C. Jnanesh A. C. Govindappa M. Murthy K. N. K. (2009). Studies on chemical weed control in aerobic rice (*Oryza sativa* L.). *J. Crop Weed* 5 321–324.

[B39] Hanzlik K. Gerowitt B. (2012). Occurrence and distribution of important weed species in German winter oilseed rape fields. *J. Plant Dis. Prot.* 119 107–120. 10.1007/BF03356429

[B40] Hikosaka K. Onoda Y. Kinugasa T. Nagashima H. Anten N. P. R. Hirose T. (2005). Plant responses to elevated CO2 concentration at different scales: leaf, whole plant, canopy, and population. *Ecol. Res.* 20 243–253. 10.1007/s11284-005-0041-1

[B41] Holm L. G. Doll J. Holm E. Pancho J. Herverger J. (1997). *Worlds Weeds: Natural Histories and Distribution*. New York, NY: John Wiley & Sons, 1129.

[B42] Howden S. M. Soussana J. F. Tubiello F. N. Chhetri N. Dunlop M. Meinke H. (2007). Adapting agriculture to climate change. *Proc. Natl. Acad. Sci. U.S.A.* 104 19691–19696. 10.1073/pnas.0701890104 18077402 PMC2148359

[B43] Hyvonen T. (2011). Impact of temperature and germination time on the success of a C4 weed in a C3 crop. *Amaranthus retroflexus* and spring barley. *Agric. Food Sci.* 20 183–190. 10.2137/145960611797215664

[B44] Idso C. D. Idso S. B. Balling R. C. Jr. (1988). The urban CO2 dome of Phoenix, Arizona. *Physical. Geogr.* 19 95–108.

[B45] Idso C. D. Idso S. B. Balling R. C. Jr. (2001). An intensive two week study of an urban CO2 dome in Phoenix Arizona, USA. *Atmos. Environ.* 35 995–1000. 10.1016/S1352-2310(00)00412-X

[B46] IPCC (1995). *Climate Change: A Glossary by the Intergovernmental Panel on Climate Change*. Geneva: IPPCC.

[B47] IPCC (2007). *Climate Change: Impacts, Adaptation and Vulnerability*. Geneva: IPCC Secretariat, 986.

[B48] Jaganathan G. K. Liu B. (2015). Role of seed sowing time and microclimate on germination and seedling establishment of *Dodonaea viscosa* (Sapindaceae) in a seasonal dry tropical environment — an insight into restoration efforts. *Botany* 93 23–29.

[B49] Johnson B. C. Young B. G. (2002). Influence of temperature and relative humidity on the foliar activity of mesotrione. *Weed Sci.* 50 157–161. 10.1614/0043-17452002050[0157:IOTARH]2.0.CO;2

[B50] Kang M. S. Banga S. S. (2013). “Global agriculture and climate change: a perspective,” in *Combating Climate Change: An Agricultural Perspective*, eds Kang M. S. Banga S. S. (Boca Raton, FL: CRC Press), 11–25.

[B51] Keeling C. D. Whorf T. P. (2004). “Atmospheric CO_2_ records from sites in the SIO air sampling network,” in *Trends 93: A Compendium of Data on Global Change. Carbon Dioxide Information Analysis Center*, eds Boden T. A. Kaiser D. P. Sepanski R. J. Stoss F. W. (Oak Ridge, TN: Oak Ridge National Laboratory, U.S. Department of Energy).

[B52] Killman W. (2008). “Foreword,” in *Climate Change and Food Security: A Framework Document*. Rome: FAO of the United Nations, iii.

[B53] Kimbal B. A. (1983). Carbon diooxide and agricultural yield. An assemblage and analysis of 430 prior observations. *Agron. J.* 75 779–788. 10.2134/agronj1983.00021962007500050014x

[B54] Knapp A. K. Hamerlyn C. K. Owensby C. E. (1993). Photosynthetic and water relations response to elevated CO2 in the C4 grass, *Andropogon gerardii*. *Int. J. Plant Sci.* 154 459–466. 10.1086/297129

[B55] Koizumi H. Kibe T. Nakada T. Yazaki Y. Adachi M. Inatomi M. (2004). “Effect of free-air CO 2 enrichment on structures of weed communities and CO 2 exchange at the flood-water surface in a rice paddy field,” in *Global Environmental Change in the Ocean and on Land*, eds Shiyomi M. Kawahata H. Koizumi H. Tsuda A. Awaya Y. 473–485.

[B56] Kudsk P. Kristensen J. L. (1992). “Effect of environmental factors on herbicide performance,” in *Proceedings of the First International Weed Control Congress*, Melbourne, VIV, 173–186.

[B57] Kumaratilake A. R. Preston C. (2005). Low temperature reduces glufosinate activity and translocation in wild radish (*Raphanus raphanistrum*). *Weed Sci.* 53 10–16. 10.1614/WS-03-140R

[B58] Latif M. A. Islam M. R. Ali M. Y. Saeque M. A. (2005). Validation of the system of rice intensification (SRI) in Bangladesh. *Field Crops Res.* 93 281–292. 10.1016/j.fcr.2004.10.005

[B59] Lee J. (2011). Combined effect of elevated CO2 and temperature on the growth and phenology of two annual C3 and C4 weedy species. *Agric. Ecosys. Environ.* 140 484–491. 10.1016/j.agee.2011.01.013

[B60] Lima M. Navarrete L. González-Andujar J. L. (2012). Climate effects and feedback structure determining weed population dynamics in a long-term experiment. *PLoS ONE* 7:e30569 10.1371/journal.pone.0030569 PMC3260292 22272362

[B61] Mahajan G. Singh S. Chauhan B. S. (2012). Impact of climate change on weeds in the rice-wheat cropping system. *Curr. Sci.* 102 1254–1255.

[B62] Mahan J. R. Dotray P. A. Light G. G. (2004). Thermal dependence of enzyme function and inhibition; implications for, herbicide efficacy and tolerance. *Physiol. Plant.* 20 187–195. 10.1111/j.0031-9317.2004.0255.x 15032852

[B63] Manea A. Leishman M. R. Downey P. O. (2011). Exotic C4 grasses have increased tolerance to glyphosate under elevated carbon dioxide. *Weed Sci.* 59 28–36. 10.1614/WS-D-10-00080.1

[B64] Matloob A. Khaliq A. Chauhan B. S. (2015a). Weeds of rice in Asia: problems and opportunities. *Adv. Agron.* 130 291–336. 10.1016/bs.agron.2014.10.003

[B65] Matloob A. Khaliq A. Tanveer A. Husssain S. Aslam F. Chauhan B. S. (2015b). Weed dynamics in dry direct-seeded fine rice as influenced by tillage system, sowing time and weed competition duration. *Crop Prot.* 71 25–38. 10.1016/j.cropro.2015.01.009

[B66] McDonald A. Riha S. DiTommaso A. DeGaetano A. (2009). Climate change and the geography of weed damage: analysis of U.S. maize systems suggests the potential for significant range transformations. *Agric. Ecosys. Environ.* 130 131–140. 10.1016/j.agee.2008.12.007

[B67] Mcdowell R. W. Condron L. M. Main B. E. Dastgheib F. (1997). Dissipation of imazapyr, flumetsulam and thifensulfuron in soil. *Weed Res.* 37 381–389. 10.1046/j.1365-3180.1997.d01-73.x

[B68] Mohamed K. I. Bolin J. F. Musselman L. J. Peterson T. A. (2007). “Genetic diversity of *Striga* and implications for control and modelling future distributions,” in *Integrating New Technologies for Striga Control–Towards Ending the Witch-Hunt*, eds Ejeta G. Gressel J. (Singapore: World Scientific), 71–84.

[B69] Mohamed K. I. Papes M. Williams R. Benz B. W. Peterson T. A. (2006). Global invasive potential of 10 parasitic witchweeds related Orobanchaceae. *Ambio* 35 281–288. 10.1579/05-R-051R.1 17240760

[B70] Mooney H. A. Hobbs R. J. (2000). *Invasive Species in a Changing World.* Washington, DC: Island Press, 457.

[B71] Mortensen D. A. Coble H. D. (1989). The influence of soil water content on common cocklebur (*Xanthium strumarium*) interference in soybeans (*Glycine max*). *Weed Sci.* 37 76–83.

[B72] Nichols V. Verhulst N. Cox R. Govaerts B. (2015). Weed dynamics and conservation agriculture principles: a review. *Field Crops Res.* 183 56–68. 10.1016/j.fcr.2015.07.012

[B73] Nowak R. S. Ellsworth D. S. Smith S. D. (2004). Functional responses of plants to elevated atmospheric CO2: do photosynthetic and productivity data from FACE experiments support early predictions? *New Phytol.* 162 253–280. 10.1111/j.1469-8137.2004.01033.x

[B74] Ooi M. K. J. Denham A. J. Santana V. M. Auld T. D. (2014). Temperature thresholds of physically dormant seeds plant functional response to fire: variation among species and relative impact of climate change. *Ecol. Evolut.* 4 656–671. 10.1002/ece3.973 PMC4098144 25035805

[B75] Orlandini S. Nejedlik P. Eitzinger J. Alexandrov V. Toulios L. Calanca P. (2008). Impacts of climate change and variability on european agriculture. Results of inventory analysis in COST 734 countries. *Trends Dir. Clim. Res.* 1146 338–353. 10.1196/annals.1446.013 19076423

[B76] Parry M. Rosenzweig C. Livermore M. (2005). Climate change, global food supply and risk of hunger. *Philos. Trans. R. Soc. B* 360 2125–2138. 10.1098/rstb.2005.1751 PMC1569580 16433098

[B77] Pathak H. Tewari A. N. Sankhyan S. Dubey D. S. Mina U. Singh V. K. (2011). Direct-seeded rice: potential, performance and problems – a review. *Curr. Adv. Agric. Sci.* 3 77–88.

[B78] Patterson D. T. (1986). Responses of soybean (*Glycine max*) and three C4 grass weeds to CO2 enrichment during drought. *Weed Sci.* 34 203–210.

[B79] Patterson D. T. (1995). Weeds in a changing climate. *Weed Sci.* 43 685–701.

[B80] Patterson D. T. Flint E. P. (1982). Interacting effects of CO2 and nutrient concentration. *Weed Sci.* 30 389–394.

[B81] Patterson D. T. Highsmith M. T. (1989). Competition of spurred anoda (*Anoda cristata*) and velvet leaf (*Albutilon theophrasti*) with cotton (*Gossypium hirsutum*) during simulated drought and recovery. *Weed Sci.* 37 658–664.

[B82] Patterson D. T. Highsmith M. T. Flint E. P. (1988). Effects of temperature and CO2 concentration on the growth of cotton (*Gossypium hirsutum*), spurred anoda (*Anoda cristata*), and velvetleaf (*Abutilon theophrasti*). *Weed Sci.* 36 751–757.

[B83] Patterson D. T. Musser R. L. Flint E. P. Eplee R. E. (1982). Temperature responses and potential for spread of witchweed (*Striga lutea*) in the United States. *Weed Sci.* 30 87–93.

[B84] Patterson D. T. Westbrook J. K. Joyce R. J. V. Lingren P. D. Rogasik J. (1999). Weeds, insects, and diseases. *Climate Change* 43 711–727. 10.1023/A:1005549400875

[B85] Peters K. Breitsameter L. Gerowitt B. (2014). Impact of climate change on weeds in agriculture: a review. *Agron. Sustain. Dev.* 34 707–721. 10.1007/s13593-014-0245-2

[B86] Peters K. Gerowitt B. (2014). Important maize weeds profit in growth and reproduction from climate change conditions represented by higher temperatures and reduced humidity. *J. Appl. Bot. Food Qual.* 87 234–242.

[B87] Petit J. R. Jouzel J. Raynaud D. Barkov N. I. Barnola J. M. Basile I. (1999). Climate and atmospheric history of the past 420000 years from the Vostok ice core, Antarctica. *Nature* 399 429–436. 10.1038/20859

[B88] Phillips O. L. Martinez R. V. Arroyo L. Baker T. R. Killeen T. Lewis S. L. (2002). Increasing dominance of large lianas in Amazonian forests. *Nature* 418 770–774. 10.1038/nature00926 12181565

[B89] Phoenix G. K. Press M. C. (2005). Effects of climate change on parasitic plants: the root hemiparasitic Orobanchaceae. *Folia Geobotanica* 40 205–216. 10.1007/BF02803235

[B90] Polley H. W. Johnson H. B. Tischler C. R. (2002). Woody invasion of grasslands: evidence that CO_2_ enrichment indirectly promotes establishment of *Prosopis glandulosa*. *Plant Ecol.* 164 85–94. 10.1023/A:1021271226866

[B91] Prior S. A. Rogers H. H. Mullins G. L. Runion G. B. (2003). The effects of elevated atmospheric CO2 and soil P placement on cotton root deployment. *Plant Soil* 255 179–187. 10.1023/A:1026143410238

[B92] Ramesh K. (2015). Weed problems, ecology and management options in conservation agriculture: issues and perspectives. *Adv. Agron.* 131 251–280. 10.1016/bs.agron.2014.12.003

[B93] Ramsey R. J. L. Stephenson G. R. Hall J. C. (2005). A review of the effects of humidity, humectants, and surfactant composition on the absorption and efficacy of highly water-soluble herbicides. *Pesticide Biochem. Physiol.* 82 162–175. 10.1016/j.pestbp.2005.02.005

[B94] Reddy P. P. (2013). Impact of climate change on insect pests, pathogens and nematodes. *Pest. Manag. Hortic. Ecosys.* 19 225–233.

[B95] Ritter R. L. Coble H. D. (1981). Influence of temperature and relative humidity on the activity of acifluorfen. *Weed Sci.* 29 480–485.

[B96] Rodenburg J. Meinke H. Johnson D. E. (2011). Challenges for weed management in African rice systems in a changing climate. *J. Agric. Sci.* 149 427–435. 10.1017/S0021859611000207

[B97] Rodenburg J. Riches C. R. Kayeke J. M. (2010). Addressing current and future problems of parasitic weeds in rice. *Crop Prot.* 29 210–221. 10.1016/j.cropro.2009.10.015

[B98] Rosenzweig C. Hillel D. (1998). *Climate Change and the Global Harvest.* Oxford: Oxford University Press, 1–324.

[B99] Saito K. (2010). Weed pressure level and the correlation between weed competitiveness and rice yield without weed competition: an analysis of empirical data. *Field Crops Res.* 117 1–8. 10.1016/j.fcr.2010.02.009

[B100] Sandhu N. Jain S. Battan K. R. Jain R. K. (2012). Aerobic rice genotypes displayed greater adaptation to water-limited cultivation and tolerance to polyethyleneglycol-6000 induced stress. *Physiol. Mol. Biol. Plants* 18 33–43. 10.1007/s12298-011-0094-2 23573038 PMC3550527

[B101] Schimel D. Alves D. Enting I. Heimann M. Joos F. Raynaud D. (1996). “Radiative forcing of climate change,” in *Climate Change 1995: The Science of Climate Change*, eds Houghton J. T. Filho L. G. M. Callander B. A. Harris N. Kattenberg A. Maskell K. (Cambridge: Cambridge University Press), 65–131.

[B102] Seck P. A. Diagne A. Mohanty S. Wopereis M. C. S. (2012). Crops that feed the world 7: rice. *Food Sec.* 4 7–24. 10.1007/s12571-012-0168-1

[B103] Shaner D. L. (2000). The impact of glyphosate-tolerant crops on the use of other herbicides and on resistance management. *Pest Manage. Sci.* 56 320–326. 10.1002/(SICI)1526-4998(200004)56:4<320::AID-PS125<3.0.CO;2-B

[B104] Sharma S. D. Singh M. (2001). Environmental factors affecting absorption and bio-efficacy of glyphosate in Florida beggar weed (*Desmodium tortuosum*). *Crop Prot.* 20 511–516. 10.1016/S0261-2194(01)00065-5

[B105] Sinha S. K. Swaminathan M. S. (1991). Deforestation, climate change and sustainable nutri tion security: a case study of India. *Clim. Change* 19 201–209. 10.1007/BF00142227

[B106] Soomro B. (2004). Paddy and water environment related issues, problems and prospects in Pakistan. *Paddy Water Environ.* 2 41–44. 10.1007/s10333-004-0045-4

[B107] Springer C. J. Ward J. K. (2007). Flowering time and elevated atmospheric CO2. *New Phytol.* 176 243–255. 10.1111/j.1469-8137.2007.02196.x 17822407

[B108] Stratonovitch P. Storkey J. Semenov M. A. (2012). A process-based approach to modelling impacts of climate change on the damage niche of an agricultural weed. *Glob. Change Biol.* 18 2071–2080. 10.1111/j.1365-2486.2012.02650.x

[B109] Treharne K. (1989). “The implications of the ‘greenhouse effect’ for fertilizers and agrochemicals,” in *The Greenhouse Effect and UK Agriculture, CAS Paper 19*, ed. Bennet R. C. (Reading: University. of Reading Press), 67–78.

[B110] Tremmel D. C. Patterson D. T. (1993). Responses of soybean and five weeds to CO2 enrichment under two temperature regimes. *Can. J. Plant Sci.* 73 1249–1260. 10.4141/cjps93-164

[B111] Tubiello F. N. Soussana J. F. Howden S. M. (2007). Crop and pasture response to climate change. *Proc. Natl. Acad. Sci. U.S.A.* 104 19686–19690. 10.1073/pnas.0701728104 18077401 PMC2148358

[B112] Tylianakis J. M. Didham R. K. Bascompte J. Wardle D. A. (2008). Global change and species interactions in terrestrial ecosystems. *Ecol. Lett.* 11 1351–1363. 10.1111/j.1461-0248.2008.01250.x 19062363

[B113] Tyree M. T. Alexander J. D. (1993). Plant water relations and the effects of elevated CO2: a review and suggestions for future research. *Vegetation* 10 47–62. 10.1007/BF00048144

[B114] Walck J. L. Hidayati S. N. Dixon K. W. Thompson K. Poschlod P. (2011). Climate change and plant regeneration from seed. *Glob. Change Biol.* 17 2145–2161. 10.1111/j.1365-2486.2010.02368.x

[B115] Wand S. J. Midgley G. F. Jones M. H. Curtis P. S. (1999). Response of wild C4 and C3 (Poaceae) species to elevated atmospheric CO2 concentration: a meta analytic test of current theories and perceptions. *Glob. Change Biol.* 5 723–741. 10.1046/j.1365-2486.1999.00265.x

[B116] Ward J. K. Tissue D. T. Thomas R. B. Strain B. R. (1999). Comparative responses of model C3 and C4 plants to drought in low and elevated CO2. *Glob. Change Biol.* 5 857–867. 10.1046/j.1365-2486.1999.00270.x

[B117] Weber E. Gut D. (2005). A survey of weeds that are increasingly spreading in Europe. *Agron. Sustain. Dev.* 25 109–121. 10.1051/agro:2004061

[B118] Wolfe D. W. Ziska L. Petzoldt C. Seaman A. Chase L. Hayhoe K. (2008). Projected change in climate thresholds in the North eastern U.S.: implications for crops, pests, livestock, and farmers. *Mitigat. Adapt. Strat. Glob. Change* 13 555–575. 10.1007/s11027-007-9125-2

[B119] World Meteorological Organization (1992). *International Meteorological Vocabulary*, 2nd Edn Geneva: World Meteorological Organization.

[B120] Zhu C. Zeng Q. Ziska L. H. Zhu J. Xie Z. Liu G. (2008). Effect of nitrogen supply on carbon dioxide induced changes in competition between rice and barnyard grass (*Echinochloa crus-galli*). *Weed Sci.* 56 66–71. 10.1614/WS-07-088.1

[B121] Ziska H. Ghannoum O. Baker J. T. Conroy J. Bunce J. A. Kobayashi K. (2001). A global perspective of ground level, ‘ambient’ carbon dioxide for assessing the response of plants to atmospheric CO2. *Glob. Change Biol.* 7 789–796. 10.1111/j.1365-2486.2001.00436.x

[B122] Ziska L. H. (2000). The impact of elevated CO2 on yield loss from a C3 and C4 weed in field-grown soybean. *Glob. Change Biol.* 6 899–905. 10.1046/j.1365-2486.2000.00364.x

[B123] Ziska L. H. (2001). Changes in competitive ability between a C4 crop and a C3 weed with elevated carbon dioxide. *Weed Sci.* 49 622–627. 10.1614/0043-17452001049[0622:CICABA]2.0.CO;2

[B124] Ziska L. H. (2004). “Rising carbon dioxide and weed ecology,” in *Weed Biology and Management*, ed. Inderjit (Dordrecht: Kluwer Publishing), 159–176.

[B125] Ziska L. H. (2008). Rising atmospheric carbon dioxide and plant biology: the overlooked paradigm. *DNA Cell Biol.* 27 165–172. 10.1089/dna.2007.0726 18318648

[B126] Ziska L. H. Blumenthal D. M. Runion G. B. Hunt E. R. Jr. Diaz-Soltero H. (2011). Invasive species and climate change: an agronomic perspective. *Clim. Change* 105 13–42. 10.1371/journal.pone.0081510

[B127] Ziska L. H. Dukes J. S. (2011). *Weed Biology and Climate Change.* Ames, IA: Blackwell Publishing Ltd, 68–205.

[B128] Ziska L. H. Faulkner S. S. Lydon J. (2004). Changes in biomass and root : shoot ratio of field-grown Canada thistle (*Cirsium arvense*), a noxious, invasive weed, with elevated CO2: implications for control with glyphosate. *Weed Sci.* 52 584–588. 10.1614/WS-03-161R

[B129] Ziska L. H. George K. (2004). Rising carbon dioxide and invasive, noxious plants: potential threats and consequences. *World Res. Rev.* 16 427–447.

[B130] Ziska L. H. Goins E. W. (2006). Elevated atmospheric carbon dioxide and weed populations in glyphosate treated soybean. *Crop Sci.* 46 1354–1359. 10.2135/cropsci2005.10-0378

[B131] Ziska L. H. McClung A. (2008). Differential response of cultivated and weedy (red) rice to recent and projected increases in atmospheric carbon dioxide. *Agron. J.* 100 1259–1263. 10.1371/journal.pone.0037522

[B132] Ziska L. H. McConnell L. L. (2015). Climate change, carbon dioxide, and pest biology: monitor, mitigate, management. *J. Agric. Food Chem.* 64 6–12. 10.1021/jf506101h 25671793

[B133] Ziska L. H. Teasdale J. R. (2000). Sustained growth and increased tolerance to glyphosate observed in a C3 perennial weed, quackgrass (*Elytrigia repens*), grown at elevated carbon dioxide. *Aust. J. Plant Physiol.* 27 159–164.

[B134] Ziska L. H. Teasdale J. R. Bunce J. A. (1999). Future atmospheric carbon dioxide may increase tolerance to glyphosate. *Weed Sci.* 47 608–6015.

[B135] Ziska L. H. Tomecek M. B. Gealy D. R. (2010). Competitive interactions between cultivated and red rice as a function of recent and projected increases in atmospheric carbon dioxide. *Agron. J.* 102 118–123. 10.2134/agronj2009.0205

